# Supplementary material for: H2A.Z is dispensable for both basal and activated transcription in post-mitotic mouse muscles
Source: Nucleic Acids Res. 2020 Apr 8;48(9):4601–13. doi: 10.1093/nar/gkaa157 (PMC7229818; doi:10.1093/nar/gkaa157)
Supplement: gkaa157_Supplemental_Files [file gkaa157_supplemental_files.zip › Supplementary_Material_-_Legends_-figures_-Tables.pdf]

SUPPLEMENTARY INFORMATIONS

Supplementary materials and methods

Histology analysis

Serial cross-sectioned (10µm thick) were collected along the entire length of embedded muscle tissues. Four consecutive slices were stained for following histological and histochemical analysis: HE, Gomori's Trichrome (TRICH), cytochrome oxidase (COX), succinate dehydrogenase (SDH), and reduced nicotinamide adenine dinucleotide tetrazolium reductase (NADH-TR). Staining was done according to standard protocols.

Immunofluorescence

For myofibers staining, an EDL muscle was collected and fixed in 4% paraformaldehyde 10 min for tissue dissection and staining. Muscles were slowly teased with fine forceps to isolate individual fibers and small fiber bundles. Fibers were permeabilized in 1X PBS, 0.1% Triton for 30 min at room temperature, saturated in 1X PBS 1% BSA for 1 hour before overnight incubation with the primary antibody (rabbit home-made anti-H2A.Z 1/100 and mouse anti-PAX7 AB_528428 from DSHB) diluted in blocking buffer. After washing, fibers were incubated with a rabbit secondary antibody conjugated with FITC and mouse secondary antibody conjugated with Alexa-555 (Molecular probes). DNA was stained with DAPI (2 µg/ml) for 2 hours at room temperature. After washing, fibers were mounted on glass slide with Vectashield (Vector Laboratories) and analysed using a Andor Spinning Disk microscope. Images were processed using ImageJ software.

Supplementary Figures

Supplementary Figure 1: Immunofluorescence for H2A.Z (in green) and PAX7 (in red) on *tibialis anterior* muscle fibers from 4 weeks old CTL mice. DAPI was used to stain DNA. White arrows mark myonuclei of the muscle fiber, orange arrows mark satellite cells (Scale bar 10 µm).

Supplementary Figure 2: Read sum up obtain from the RNA-seq of H2A.Z dKO muscle obtain around each H2A.Z genes. Note that reads are present only in the beginning of each H2A.Z genes and not in the regions located between loxP sites (end of each genes).

Supplementary Figure 3: RT-qPCR analyses of genes found up-regulated (**A**) and down-regulated (**B**) by RNA-seq performed on control (CTL) and H2A.Z1/H2A.Z2 KO (H2A.Z dKO) TA muscles (n = 3).

Supplementary Figure 4: RT-qPCR analyses of MyoD (**A**) and Myogenin (**B**) after denervation of TA muscle in presence (CTL) or absence of H2A.Z (H2A.Z dKO).

Supplementary Figure 5: Phenotypic characterisation of control and H2A.Z dKO mice in innervated and denervated conditions after 48h. **(A)** Transverse sections of TA muscle were histochemically stained with Haematoxylin Eosin (HE), Gomori's Trichrome (TRICH), SDH-CoxH and NADH activity. (scale bar 500µm) **(B)** Zoom in of supplementary figure 1A with the same staining. (scale bar 100µm)

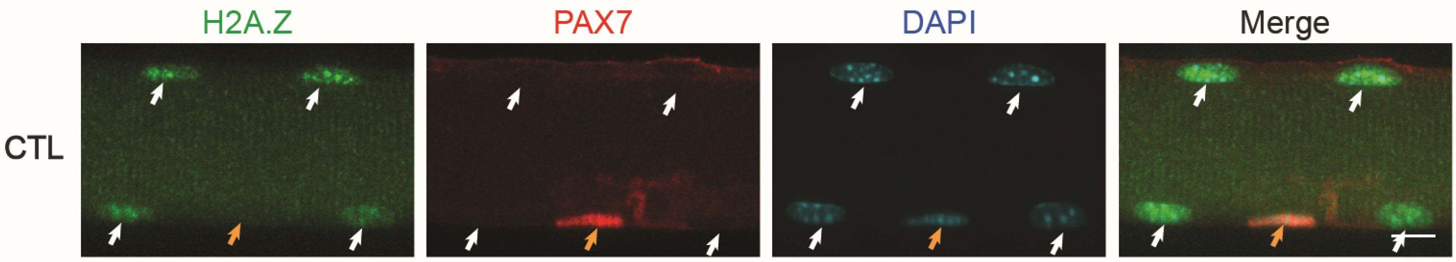

Supplementary Figure 1

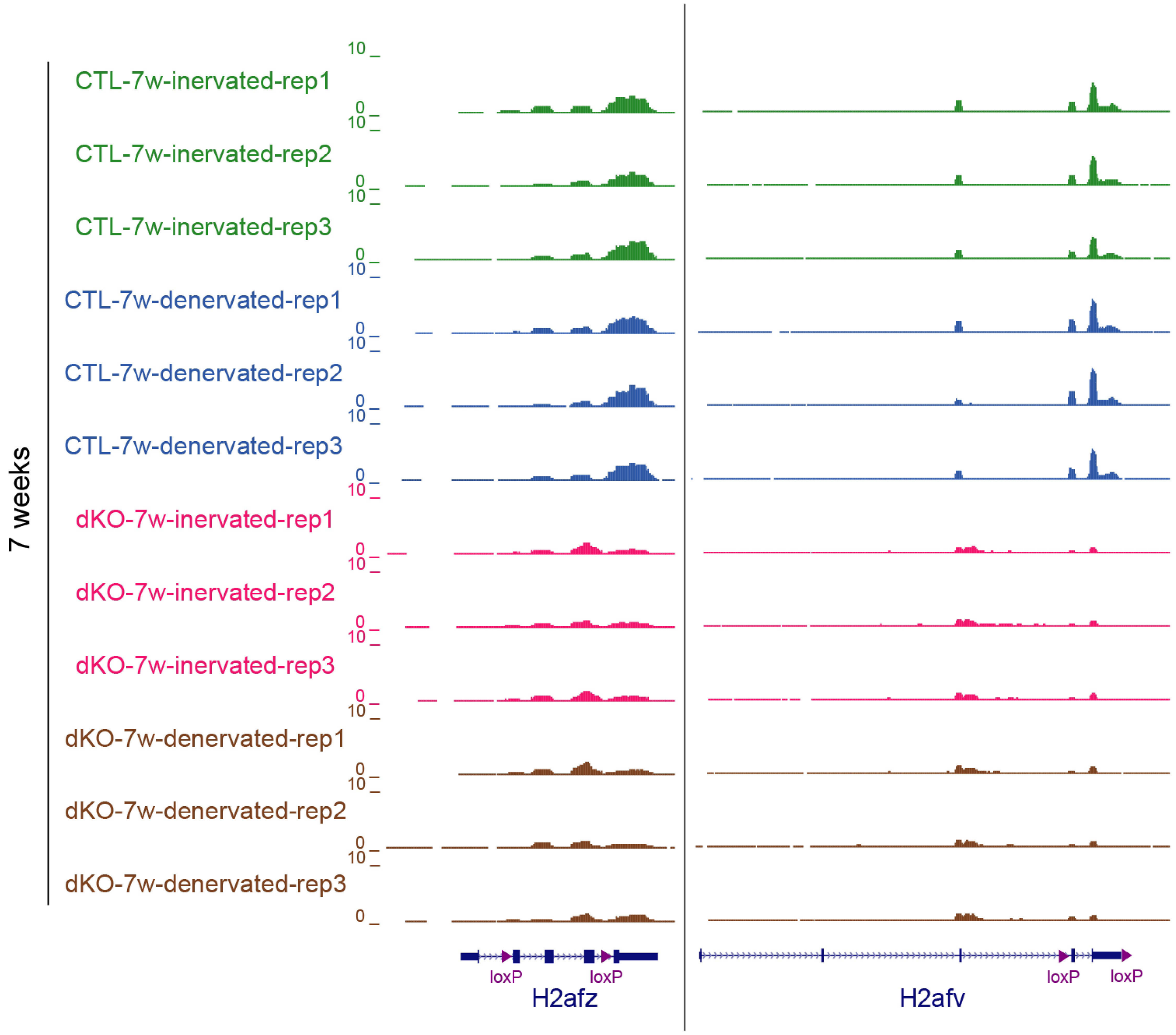

Supplementary Figure 2

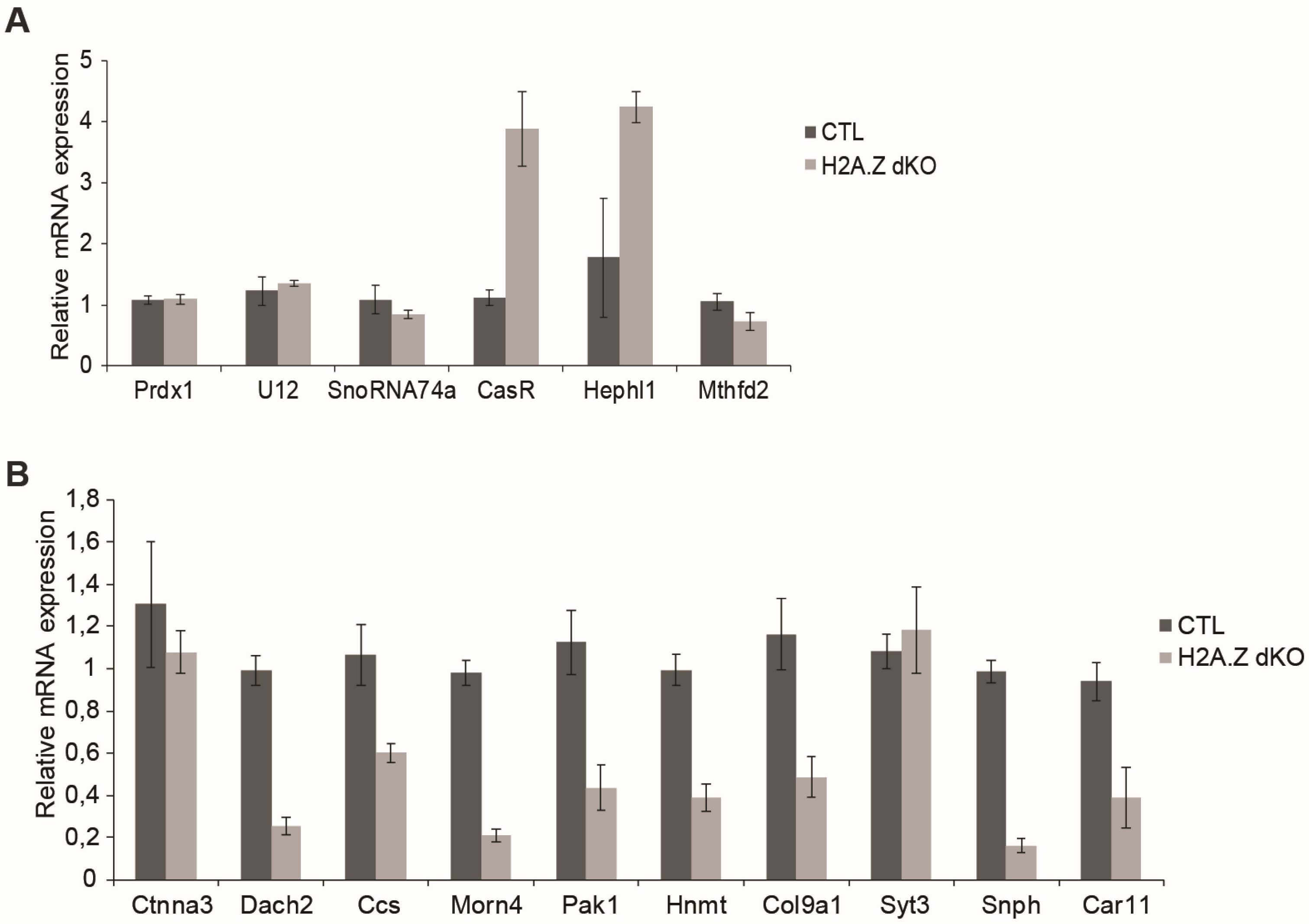

Supplementary Figure 3

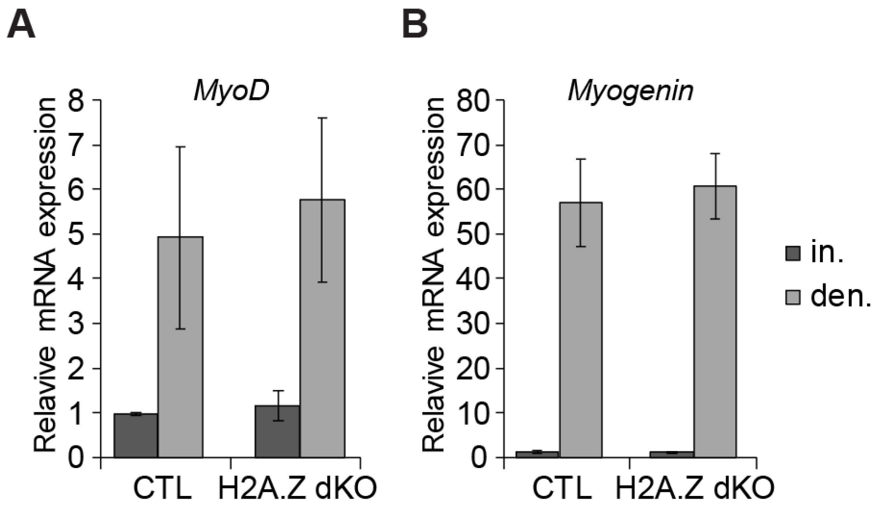

Supplementary Figure 4

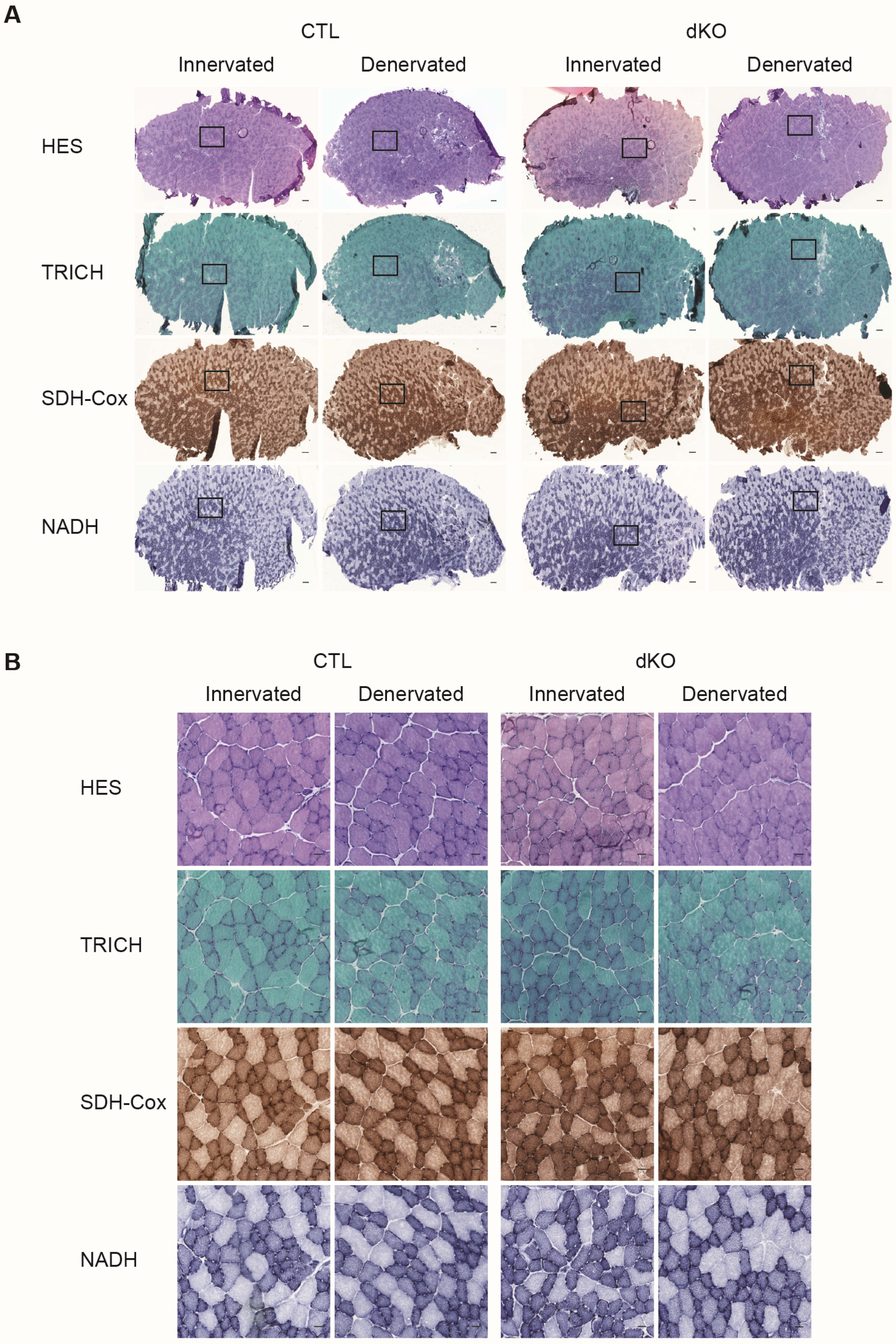

Supplementary Figure 5

Supplementary Table 1

Genotyping primers

	primer	sequence	alleles	product size (bp)
**H2afz**	Ef 4421	GCTACCATTGCTGGTGGTGGTATGTCA	WT and floxed	278
	Er 4422	TGTGTGGGATGACACCTAGGAGAGGG		388
**H2afv**	Ef 5978	CCACGTAATGAGATCCAGTGCCCT	WT and floxed	257
	Er 5981	CATGCACCTGCCATTATGTCTGGTA		433

**Cre**	F	CGATGCAACGAGTGATGAGG	Tg	288
	R	GCATTGCTGTCACTTGGTCGT		
**CD8**	F	GGTGCATTCTCACTCTGAGTTCC	endogenous control	100
	R	GCAGACAGAGCTGATTTCCTATGTG		

Supplementary table 2

Q-PCR primers

	**Forward**	**Reverse**
H2A-Z.1	AGGTAAAGCGTATCACCCCTC	CGATCAGCGATTTGTGGATGTG
H2A-Z.2	CATCACCCCACGTCACTTACA	GTGGCCTTGATAAGAGAATCCAA
RPL41	GCCATGAGAGCGAAGTGG	CTCCTGCAGGCGTCGTAG
GusB	GAGGATTGCCAACGAAACCG	GTGTCTGGGGACACCTTTGA
Myog	CAATGCACTGGAGTTCGGTC	ACAATCTCAGTTGGGCATGG
MyoD	CACGACTGCTTTCTTCACCA	CGGAACCCCAACAGTACAAT
Fut10	CTGGGCGCTTTTCCATGAAGA	TAGTCAGTGGCAAGTGGGAAT
Ociad2	GCCTGTTGTTTTGCCCCAAAT	GCGGCTAAATAACCTTGGTGG
Hecw2	GCCTGCACTCTAGGAAACTCG	TCCCTGGCTGAATGGACATCT
Stc1	CTCCAAAACTCAGCAGTGATTCT	GAGGCAGCGAACCACTTCA
Snph	TCTCCACCTGTGAGTGTGC	CTGTTCTGGGGTGGGAGG
Car11	TTATGACCCTTTTCTGCCCC	CGGTGGCTATAAAGAAGGGGA
Casr	TGGAGTAGCAGCCAAAGATCA	GTCATGTTGGGAAGAAGGGC
Prdx1	TATCAGATCCCAAGCGCACC	GAAGGCCTGGACTAGTCGTA
U12	AATAACGATTCGGGGTGACG	CAGATCGCGTCACCCAGG
SnoRNA74a	CAGTTGTCAGCTATCCAGGC	ATTGTTTGCACCCAGACCAG
Hephl1	TCTTTACTTCTCAGCGGTCCA	TGTTTTCATCAAGATAGCTGCTG
Mthfd2	CAGGAGGTGGAAGAGTGGG	CTGAGATGTGCCTGTGTCTG
Ctnna3	TCCGACCTCCAGAGAACCTA	AGGCGGATTTCTGAGTTCGA
Dach2	AGAGAAAACCTGGAGCGACA	CGCGGCGCTTTGATTCAAA
Syt3	CTTACCCAGTGCCCAGTCTC	AGAGGTAAGGGCAGTGCAG
Ccs	CAGATGGTGTTGGTGCAGAC	CTGTAGGAAGCGGACCACC
Morn4	TGGCTTTGGTCAATTGGTGT	ATGAAGACTCCGACGCCATT
Pak1	AGCAGTACCACCAGTGTCAG	TGGCGGAGTGGTGTTATTCT
Col9a1	TTTCACAGGGCAGTAGAGGC	CCTGTCTCCTTTCATTCCAGG
Hnmt	GACCAAGGCAGATACGTAGAA	CCTCCGCCTACACTCAGAAT

Supplementary Table 3. Main down- and up-regulated genes in innervated and denervated CTL and dKO H2A.Z skeletal muscle from 7 week-old mice.

Supplementary Table 4. Repetitive elements family enriched of H2A.Z. The majority of significantly H2A.Z-enriched repeats (log2 enrichment > 0.5 and P value < 10-2) belongs to the simple repeats and endogenous retrovirus classes of repetitive elements.

Supplementary Table 5. Main down- and up-regulated repetitive DNA element families in innervated and denervated CTL and dKO H2A.Z skeletal muscle from 7 week-old mice.
